# Gene-Based Burden Testing of Rare Variants in Hemiplegic Migraine: A Computational Approach to Uncover the Genetic Architecture of a Rare Brain Disorder

**DOI:** 10.3390/genes16070807

**Published:** 2025-07-09

**Authors:** Mohammed M. Alfayyadh, Neven Maksemous, Heidi G. Sutherland, Rodney A. Lea, Lyn R. Griffiths

**Affiliations:** 1 Centre for Genomics and Personalised Health, Genomics Research Centre, School of Biomedical Sciences, Queensland University of Technology (QUT), Brisbane, QLD 4059, Australia; n9261834@qut.edu.au (M.M.A.); n.maksemous@qut.edu.au (N.M.); heidi.sutherland@qut.edu.au (H.G.S.); rodney.lea@qut.edu.au (R.A.L.); 2 Central Analytical Research Facility (CARF), Faculty of Sciences, Queensland University of Technology (QUT), Brisbane, QLD 4059, Australia

**Keywords:** hemiplegic migraine, burden testing, association analysis, PathVar, bioinformatics

## Abstract

Background: HM is a rare, severe form of migraine with aura, characterised by motor weakness and strongly influenced by genetic factors affecting the brain. While pathogenic variants in *CACNA1A*, *ATP1A2*, and *SCN1A* genes have been implicated in familial HM, approximately 75% of cases lack known pathogenic variants in these genes, suggesting a more complex genetic basis. Methods: To advance our understanding of HM, we applied a variant prioritisation approach using whole-exome sequencing (WES) data from patients referred for HM diagnosis (n = 184) and utilised PathVar, a bioinformatics pipeline designed to identify pathogenic variants. Our analysis incorporated two strategies for association testing: (1) PathVar-identified single nucleotide variants (SNVs) and (2) PathVar SNVs combined with missense and rare variants. Principal component analysis (PCA) was performed to adjust for ancestral and other unknown differences between cases and controls. Results: Our results reveal a sequential reduction in the number of genes significantly associated with HM, from 20 in the first strategy to 11 in the second, which highlights the unique contribution of PathVar SNVs to the genetic architecture of HM. PathVar SNVs were more distinctive in the case cohort, suggesting a closer link to the functional changes underlying HM compared to controls. Notably, novel genes, such as *SLC38A10*, *GCOM1*, and *NXPH2*, which were previously not implicated in HM, are now associated with the disorder, advancing our understanding of its genetic basis. Conclusions: By prioritising PathVar SNVs, we identified a broader set of genes potentially contributing to HM. Given that HM is a rare condition, our findings, utilising a sample size of 184, represent a unique contribution to the field. This iterative analysis demonstrates that integrating diverse variant schemes provides a more comprehensive view of the genetic factors driving HM.

## 1. Introduction

Migraine is a severe neurovascular disorder influenced by genetic factors, characterised by intense head pain and aura [1]. The International Headache Society classifies migraines into two categories: migraine with aura (MA) and migraine without aura (MO) [2]. Hemiplegic migraine (HM), a rare and debilitating subtype of MA, marked by motor weakness on one side of the body [3], typically manifests in childhood or adolescence [4]. HM is primarily driven by genetic mutations that impair ion channel function in neurons, ultimately leading to increased neuronal excitability and the initiation of cortical spreading depression (CSD) [5,6,7]. Mutations in genes such as *CACNA1A*, *ATP1A2*, *SCN1A*, and *PRRT2* disrupt the proteins responsible for regulating the transport of key ions, such as calcium, sodium, and potassium, across neuronal membranes [7,8,9]. This ion channel dysfunction impairs proper neurotransmission and promotes the excessive depolarisation of nerve cells. As a result, neurons become abnormally excitable, setting the stage for CSD, a propagating wave of disrupted electrical activity in the brain. This phenomenon is responsible for the aura symptoms characteristic of HM, including temporary motor weakness and sensory disturbances [10]. While similar to the aura observed in a typical migraine, the symptoms in HM are more severe and occur more readily due to the underlying genetic channelopathy (e.g., *CACNA1A*, *ATP1A2*, *SCN1A*, *PRRT2*) [7].

Diagnosing HM can be challenging due to symptom overlap with other conditions such as stroke [4,11], and it affects approximately 0.01% of European populations [4,12]. HM is categorised based on family history, with familial hemiplegic migraine (FHM) linked to pathogenic variants in the *CACNA1A*, *ATP1A2*, and *SCN1A* ion transport genes [13]. HM can arise from de novo pathogenic or likely pathogenic variants. De novo pathogenic variants are genetic changes that occur spontaneously in an individual and are not inherited from either parent. These variants can play a major role in rare neurological disorders and have been implicated in a variety of neurodevelopmental diseases, including those with sporadic presentation and no family history [14]. While much of disease heritability is attributed to inherited genetic variation, de novo pathogenic variants provide an important mechanism for the occurrence of HM and other rare diseases, especially in cases where there is no prior family history. However, approximately 75% of HM patients lack mutations in these ion channel genes, suggesting additional genetic factors [15]. Notably, previous work has shown an increased burden of missense variants in other related genes, the *CACNA1H* and *CACNA1l* genes [16]. Genome-wide association studies (GWASs), which scan the genome to identify genetic variants associated with diseases or traits, primarily focus on variants with minor allele frequencies (MAFs) between 1% and 5%, but have explained only a small fraction of disease heritability, as most risk alleles identified tend to be minor alleles and the contribution of these common and uncommon variants to overall genetic risk remains limited [17,18,19]. Rare variants (MAF < 1%) may play a significant role, as they can strongly impact protein function and contribute to complex diseases [20,21,22,23,24]. Whole exome sequencing (WES) is a powerful genomic technique that sequences all the protein-coding regions (exons) of genes in the genome to identify rare or novel pathogenic variants associated with disease by specifically targeting these regions, where most disease-causing mutations occur, while GWASs primarily detects common variants and may miss rare, high-impact mutations [25,26]. It has been instrumental in identifying causal variants for diseases such as Miller syndrome [27], Kabuki syndrome [28], Alzheimer’s disease [29], and cholesterol disorders [30]. While WES does not cover non-coding regions, it remains a cost-effective method for uncovering functionally significant mutations [26]. Studying rare variants through exome sequencing holds promise for identifying additional genetic causes of complex diseases [20].

### 1.1. Rare Variant Association Tests

Investigating the role of rare variants in complex conditions remains a key challenge in genetic studies. GWASs have limited effectiveness in studying rare variants, as they primarily focus on common variants that are more prevalent in the population. Single-variant association tests for rare variants often suffer from low statistical power due to the need for large sample sizes in complex trait analyses. To address these limitations, various alternative methods have been developed to improve rare variant association testing, as summarised in Table 1 [31,32,33,34].

These approaches include collapsing (burden) tests and distribution-based analyses, which typically focus on genes or specific genomic regions. These methods group multiple rare variants, assuming that variants within a specific region may collectively influence a shared trait. Regions enriched with rare variants (MAF < 1%) that significantly affect protein function are more likely to be implicated in disease mechanisms.

Collapsing tests aggregate genetic data across predefined regions or genes into a single variable for analysis. A key example is the gene-based collapsing test, which assesses whether qualifying variants (those meeting specific criteria) are more prevalent in cases than in controls [35]. These methods enhance statistical power and have become more robust over time, proving crucial for identifying the genetic basis of diseases [33,36,37].

Various statistical approaches have been developed to assess associations between rare variants and traits, including kernel-based methods like the Kernel-Based Adaptive Cluster (KBAC) and the Sequence Kernel Association Test (SKAT). Among these, SKAT and burden tests are widely used due to their flexibility and high statistical power. SKAT, a regression-based method, is computationally efficient and allows for covariate adjustments within the model.

Burden tests aggregate data from multiple variants into a single score, considering factors such as MAF and frequency thresholds, based on the assumption that rare variants collectively increase disease risk [38]. Some methods integrate both common and rare variants, such as the Combined Multivariate and Collapsing (CMC) method, which aggregates genetic information within a gene or region and uses Hotelling’s *t*-test for case–control studies [36].

A key limitation of burden tests is the assumption that all variants affect the trait in the same direction [39,40]. These methods lose power when variants have bidirectional effects or when many variants are non-causal. Despite these challenges, rare variant association methods remain widely applied, highlighting the importance of tailoring study designs and analytical strategies for effective rare variant testing [41].

Analysing the distribution of effects from multiple variants within a genomic region is a method used for rare variant association testing. Models such as SKAT [42], C-alpha [31], and the sum of squared score (SSU) test [43] address challenges related to directionality and variability. However, these models’ power decreases when there are more causal variants or when variants lack bidirectional effects. To address limitations in variance-component or burden tests, combined *p*-values from both methods are used, leading to the development of the SKAT-O omnibus test, which integrates SKAT and burden tests using a Fisher statistic model [40,44,45]. While SKAT-O generally outperforms individual tests, it may lose power when fewer trait-associated variants are present [46,47]. The aggregated Cauchy association test (ACAT) enhances power when few causal variants are present by transforming *p*-values into Cauchy variables [48]. Gene- or region-based approaches are more powerful than single-variant methods but they are less effective when only a small number of variants are associated or when non-causal variants dominate. Gene-based collapsing tests also face challenges due to variations in genetic sub-region tolerance for missense variants, as disease-causing variants often cluster in sub-regions intolerant to genetic variation. Potential solutions include collapsing variants within specific sub-regions or adding missense intolerance as an additional filter [49,50].

### 1.2. Population Stratification

Principal Component Analysis (PCA) is a crucial tool in genetic studies, essential for understanding genetic variation and population stratification. By simplifying complex genetic data, PCA reveals patterns and correlations, aiding in the visualisation of genetic relationships between individuals and populations [51,52]. It has been instrumental in understanding ancestral origins and human migration patterns [53] and is vital in GWASs to mitigate population stratification effects that can cause spurious variant-disease associations [51]. PCA’s versatility extends to rare variant studies, where it helps detect subtle patterns [54], predicts missing variants, and identifies outlier samples [55]. Despite limitations like sensitivity to uneven sampling and SNP ascertainment biases [56], advanced PCA techniques such as robust, sparse, and kernel PCA address these challenges [57]. Overall, PCA remains a key tool in genetic research, helping to decode the complexities of human genetics [58].

In the context of HM, approximately two-thirds of patients do not have known pathogenic variants in known FHM genes [15,59], indicating that its genetic basis may be more complex than initially thought. Our previous work developed PathVar, a bioinformatics pipeline designed to identify pathogenic variants in Next-Generation Sequencing (NGS) data [60]. This pipeline enabled the identification of multiple deleterious variants and candidate genes. In the present study, we expand upon these findings by applying rare variant burden testing, a statistical method that evaluates whether the accumulation of rare, potentially damaging variants in a gene (or set of genes) is more frequent in HM patients than in controls. This method is particularly suited to complex disorders like HM, where individual rare variants may be insufficiently powered to show association on their own. By aggregating their effects at the gene level, burden testing allows us to assess the cumulative impact of these variants on disease risk. Through this approach, we aim to better characterise the genetic architecture of HM and identify genes contributing to its heritability.

## 2. Materials and Methods

### 2.1. Study Cohorts

The Genomic Research Centre (GRC) at Queensland University Technology (QUT) has previously conducted targeted gene sequencing [61] and a comprehensive WES analysis [15] on a cohort of 208 HM samples, including 187 HM cases and 21 extended family members from nine cases. The HM cases were negative for pathogenic variants in known FHM genes, including *CACNA1A*, *ATP1A2*, and *SCN1A* [15]. The WES analysis identified three cases with causal pathogenic variants, specifically two in the *ATP1A3* gene and one in *SLC1A2*, which were subsequently excluded from further analysis. Clinical data beyond the HM diagnosis are limited for this cohort. The mean age of participants was 32.9 years, with a gender distribution of 77 males and 131 females [59].

For over two decades, the GRC has been conducting National Association of Testing Authorities (NATA)-accredited diagnostic genetic testing for the primary FHM genes, with patients referred by specialist neurologists following informed consent. The demographic characteristics of this cohort, including age and sex, have been detailed in a previous publication [15]. Notably, this HM cohort is one of the largest globally, which enhances the generalisability of our findings to the broader HM patient population. The size and scope of this cohort provide a unique opportunity to investigate the genetic underpinnings of HM, and our research aims to contribute to a better understanding of this complex disorder.

For the purpose of establishing a comparative control group in our study, we utilised a total of 1035 control samples from the United Kingdom Biobank (UKBB), project number 86,460 [62]. These control samples were meticulously curated by a specialised team of statisticians and bioinformaticians at the GRC using the DNA Nexus platform. The control cohort consisted of Whole Genome Sequencing (WGS) data in the Binary Alignment Map (BAM) format, which were subsequently subset to the same exonic regions as the HM BAM files, using an Ion Torrent platform Browser Extensible Data (BED) file of exonic regions.

The control group comprised healthy individuals, defined by the absence of any diagnosed neurological conditions. Notably, this cohort displayed a well-balanced sex distribution, with an equal representation of males and females. The mean age of these samples was 55 years. The inclusion of these control samples provided a robust baseline for comparative analysis, enabling the identification of genetic associations and patterns pertinent to the study of HM.

### 2.2. Qualifying SNVs Selection

**Figure 1:**
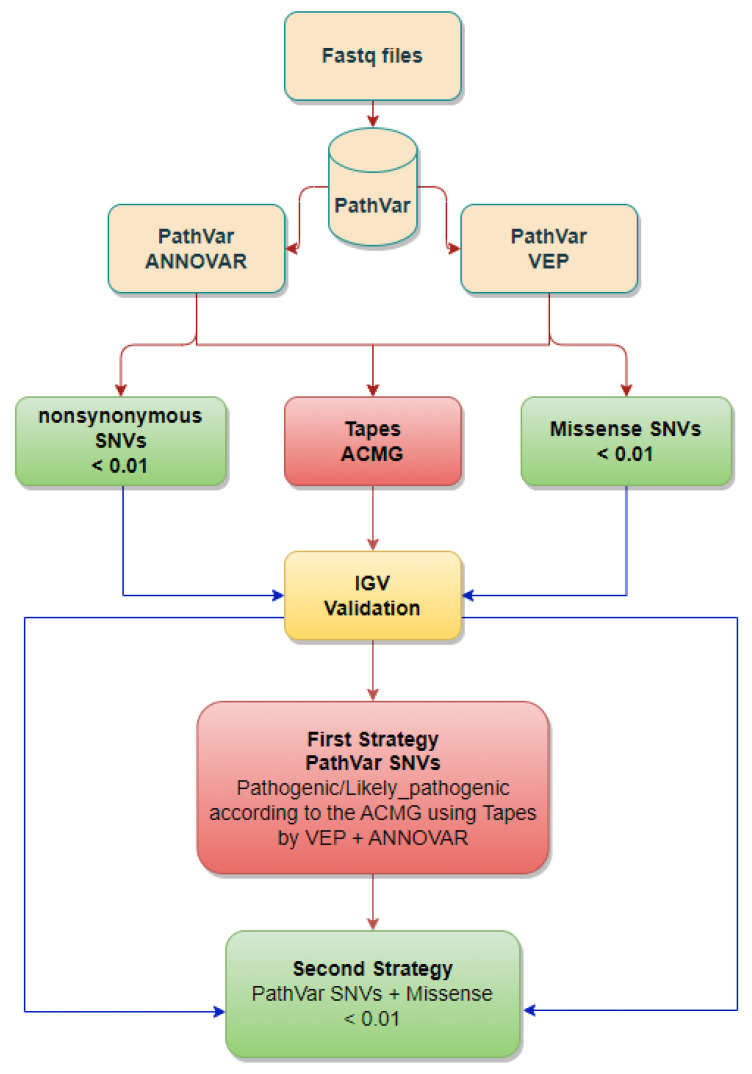
Bioinformatics pipeline for identifying and prioritising rare, potentially pathogenic SNVs from exome/genome sequencing. Sequencing data (FASTQ files) are processed using **PathVar**, a custom SNV detection tool that integrates public variant detection and annotation tools. Variants are annotated with **ANNOVAR** (gene-based and frequency data) and **VEP** (predicts functional transcript effects). Filtering retains rare nonsynonymous (ANNOVAR) and missense (VEP) SNVs with minor allele frequency (**MAF**) < 0.01 in population databases. Variants are classified by **Tapes** using **ACMG** guidelines (e.g., pathogenic, likely pathogenic). Predicted pathogenic/likely pathogenic SNVs are visually validated with **IGV**. Two prioritisation strategies are applied: (1) retain SNVs classified as pathogenic/likely pathogenic; (2) include these plus all rare missense SNVs (MAF < 0.01), allowing broader inclusion for downstream analysis.

In our previous work, we used the pathogenic variant calling (PathVar) pipeline [60] to generate a list of candidate genes for burden testing. PathVar is a novel bioinformatics algorithm that integrates publicly available tools to detect single nucleotide variants (SNVs) and generate a list of candidate genes. PathVar operates through a three-stage process: variant calling, annotation and filtration, and pathogenicity assignment. The variant calling stage utilises the Genome Analysis Toolkit (GATK) tools [63], including Base Quality Score Recalibration (BQSR) and HaplotypeCaller, to produce variant call format (VCF) files. Annotation and filtration are performed using Annotate Variant (ANNOVAR) [64], Variant Effect Predictor (VEP) [65], and machine learning-based tools, such as Variant Quality Score Recalibration (VQSR), alongside GATK hard filtration. Finally, variants are classified according to ACMG criteria [66] using TAPES [67], a software tool to identify likely pathogenic variants. PathVar identifies variants classified as pathogenic or likely pathogenic by both VEP (v110.1) and ANNOVAR. By merging text files annotated with VEP and ANNOVAR using TAPES, it extracts variants with concordant pathogenic or likely pathogenic predictions, facilitating further analysis and interpretation (Figure 1). All variants were visually validated using the Integrative Genomic Viewer IGV (v2.19.4) [68]. In this study, we generated a final set of variants by selecting those that are predicted to be pathogenic or likely pathogenic by both VEP and ANNOVAR, are missense, and have a rare allele frequency (AF) of <0.01 [60]. We will refer to these as PathVar variants for the remainder of this research.

We employed a multi-faceted approach to conduct our association analysis, prioritising genes that harbor PathVar variants. Notably, several of these genes, including *GCOM1* [69], *SETX* [70], and *SLC38A10* [71], have been previously implicated in neurological conditions. To elucidate the genetic architecture underlying this association, we implemented two distinct analytical strategies. In the first strategy, PathVar SNVs served as the qualifying SNVs. In the second strategy, we expanded the list of qualifying criteria to include SNVs that are missense and rare (AF < 0.01) in the same genes. We acknowledge that other methods, such as gene methylation profiles and RNA sequencing (RNA-seq), can offer complementary, and in some cases, alternative insights into the presence and functional impact of rare pathogenic/likely pathogenic variants. While they are not direct substitutes for DNA-based sequencing, these approaches can provide additional layers of evidence that support variant interpretation. However, here we utilise DNA-based sequencing to investigate rare variants.

### 2.3. Population Stratification Investigation

One of the most challenging aspects of the case–control design is adjusting for confounding. In genetic studies, this often manifests as population structure or population stratification. An initial investigation of population stratification was performed using PCA in PLINK v1.9. Moreover, Uniform Manifold Approximation and Projection (UMAP) dimensionality reduction techniques were also used to reduce the number of principal components (PCs) and to visualise any evidence of genetic stratification. The VCF files from both cases and controls were merged to generate a joint VCF file. We then used PLINK to generate binary (.bim, fam, bed) files from the joint VCF.

To obtain a high-quality subset of SNVs for further analysis, we applied a rigorous filtering and pruning process. The initial set of 4877 SNVs was filtered based on PathVar quality metrics, which assess the reliability and accuracy of the variant calls. This step helped to remove low-quality variants that may introduce noise or bias into the analysis. Next, we used PLINK to prune the remaining SNVs for linkage disequilibrium (LD). LD pruning is a technique that removes variants in strong correlation with each other, as these can lead to redundant information and inflated type I error rates. By removing these correlated variants, we aimed to create a subset of SNVs that captures the underlying genetic structure of the population independently of the phenotype. A variance inflation factor (VIF) threshold of 2 was applied to remove variants with high LD [72,73], and both common and rare SNVs were included. This process resulted in a final set of 2501 SNVs, which were used solely to generate the final PCs for population structure correction. Python (v3.9.16) packages including pandas, numpy, umap, KMeans from sklearn. cluster, matplotlib. Pyplot, and seaborn were used to generate the UMAP figures.

Gene-based burden testing was performed using the SKAT package, version 2.2.5, within the R software (v4.0.3) framework. Specifically, SNVs were aggregated by gene to construct a genotype matrix, adhering to the prescribed format outlined in the ‘SKAT. example’ documentation. To facilitate standard logistic regression analysis, matrices were configured such that genes were represented by columns, samples by rows, and variant presence/absence was dichotomously coded (0/1). Furthermore, standard logistic regression analyses were conducted employing the statsmodels. api and numpy packages in Python. Data visualisation was achieved through the utilisation of Python packages, including pandas, numpy, seaborn, and matplotlib. pyplot. Notably, all association models incorporated covariate adjustment, comprising the top four PCs, as well as age and sex.

## 3. Results

### 3.1. SNVs and Genes Tested

We initially identified 55 qualifying PathVar SNVs, which formed the basis of the first analytical strategy (Supplementary Table S1). These were labelled as pathogenic SNVs according to the ACMG and likely pathogenic with a minimum heterozygous count of three. These 55 SNVs were selected through a separate, stricter filtering process focused on clinical interpretation and variant confidence, including manual IGV inspection, to form the list of qualifying SNVs that were included in the analysis. Although many high-quality variants were initially identified, the stringent IGV criteria we applied necessitated the exclusion of several variants. For instance, if a variant was observed in 10 individuals but appeared reliable with good sequencing depth in only 9, it was excluded if the remaining individual either lacked the variant or displayed an unreliable call. As a result, only 55 SNVs were selected, meeting the strict IGV criteria, and were thus considered PathVar SNVs.

The 55 SNVs were mapped to 40 genes, including *ACO12*, *ADAMTSL4*, *AHR*, *AMPD1*, *APC2*, *ASPA*, *ATL3*, *C12ORF57*, *CD207 CPT2*, *CTH*, *CTNS CYP24A1*, *DNA2*, *ECSIT*, *EXOSC3*, *GCOM1*, *HUNK*, *HYKK*, *KCNQ1*, *KIAA1328*, *MCCC2*, *MPO*, *NLRX1*, *NXPH2*, *OSBPL1A*, *PADI3*, *PDE6B*, *PMM2*, *POLE*, *PROKR1*, *RCN3*, *SETX*, *SLC38A10*, *SVEP1*, *TLL1*, *TMPRSS3*, *TSHR*, *TYR*, and *ZEB1*, which were assigned to distinct functional categories based on gene ontology (Supplementary Figure S1) [74]. Burden testing was performed for these genes across both analytical strategies. In the second strategy, we expanded the inclusion criteria to incorporate non-PathVar SNVs that were missense variants with an AF of <0.01. This adjustment added 135 additional SNVs to the same 40 genes tested in the first strategy, resulting in a total of 190 qualifying SNVs for further evaluation. This approach allowed for a more comprehensive assessment of potentially impactful genetic variants within the cohort.

### 3.2. Principal Component Analysis

Our initial investigation of population stratification identified the presence of genetic stratification within the genotype data of our study population (Supplementary Figures S2 and S3). This finding highlights a clear genetic differentiation within the dataset. As is standard practice in case–control study designs, both known and unknown imbalanced predictors between cases and controls must be accounted for, as these factors could potentially confound the true relationship between HM and the genes prioritised for burden testing. Therefore, it is essential to capture genetic stratification independent of disease status and adjust for it in this research.

**Figure 2:**
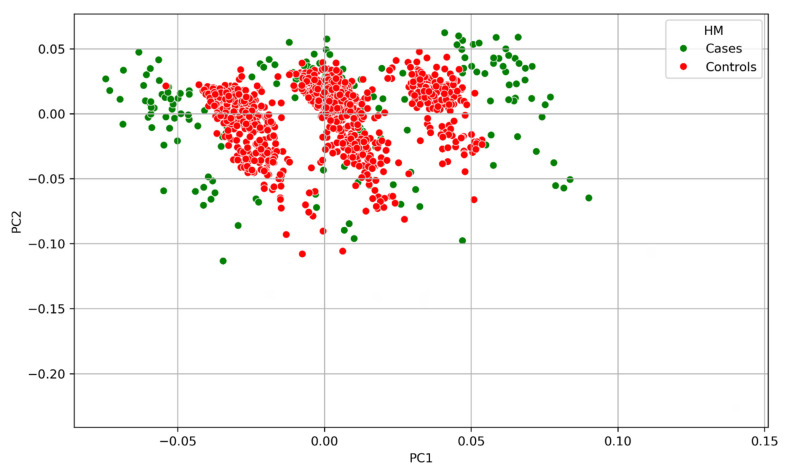
The final PCA demonstrates population stratification independent of phenotype. This is illustrated in the figure, where PC1 is plotted on the *X*-axis and PC2 on the *Y*-axis. Data points are colour-coded by phenotype: green dots represent individuals with HM, and red dots correspond to control subjects. The green and red dots’ distribution across the two PCs indicates that disease status does not influence the observed genetic clusters.

**Figure 3:**
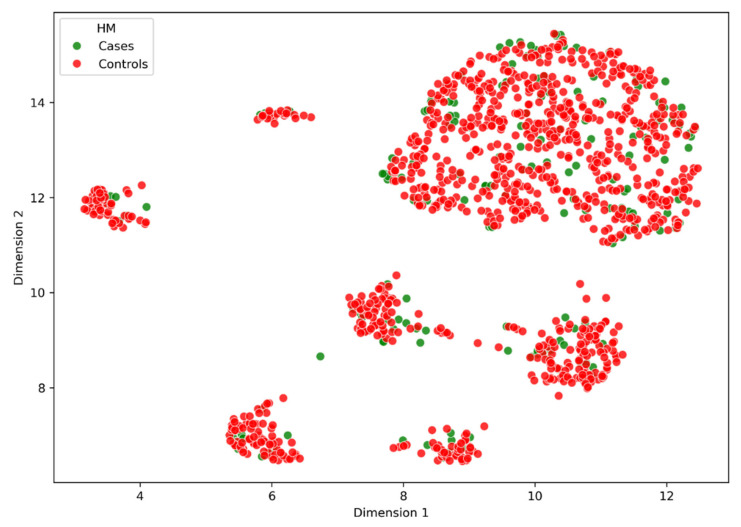
This UMAP plot provides clear evidence of genetic clusters independent of disease status. The figure represents the dimensionality reduction of 20 PCs into two dimensions, with the first dimension plotted on the *X*-axis and the second on the *Y*-axis. Data points are colour-coded according to phenotype: green dots indicate individuals with HM, while red dots represent the control cohort.

The final subset of SNVs used, comprising both rare and common variants, effectively captured the genetic stratification independent of the phenotype, as illustrated in Figure 2 and Figure 3. These figures demonstrate that the selected SNVs were able to distinguish between different population groups, indicating that they are informative about the underlying genetic differences of the individuals. The final subset of SNVs provides a robust and reliable set of markers for further analysis, allowing us to investigate the genetic associations with HM while minimising the impact of population stratification and other sources of bias.

### 3.3. Association Analysis

**Figure 4:**
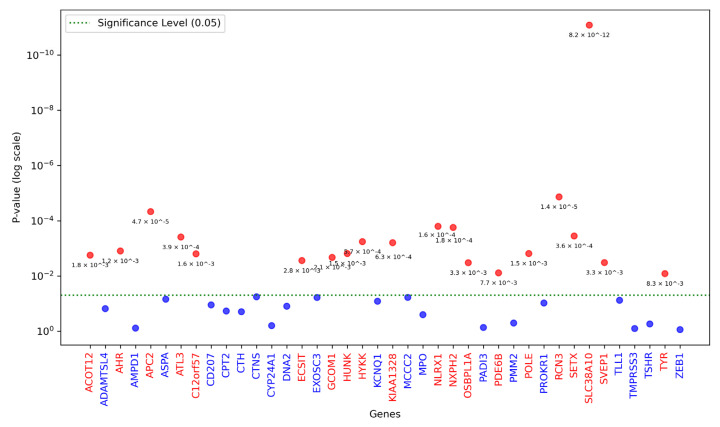
This figure illustrates the results of the burden testing analysis. PathVar genes were modelled against the phenotype column using the SKAT package in R. Only PathVar SNVs were included in this analysis. The *Y*-axis represents the *p*-value on a logarithmic scale, while the *X*-axis represents the genes. The dotted line indicates the significance threshold, with red dots corresponding to significant *p*-values and blue dots representing non-significant *p*-values. Significant genes are shown in red, while non-significant genes are depicted in blue.

Our initial strategy for performing association analysis involved conducting burden testing using PathVar SNVs as the qualifying variants. The burden-testing results indicated that several genes (20 genes) were significantly associated with the phenotype column after correcting for multiple testing using the Benjamini–Hochberg procedure false discover rate (FDR) (Figure 4), and may potentially contribute to HM. These genes exhibit a higher burden of PathVar SNVs in the case cohort compared to the control cohort after adjusting for covariates and the top four PCs. This result is expected, as PathVar variants are predicted to be either pathogenic or likely pathogenic according to the ACMG criteria, and their prevalence may be higher in the disease cohort.

In the second strategy of our analysis, we extended the list of qualifying SNVs to encompass PathVar SNVs and non-PathVar but missense SNVs with AF < 0.01. This expansion led to a reduction in the number of significant genes after adjusting for covariates. Among the 20 genes, 11 were rendered statistically insignificant, including *SVEP1*, *SETX*, *PDE6B*, *OSBPL1A*, *NLRX1*, *KIAA1328*, *HYKK*, *HUNK*, *C12orf57*, *APC2*, and *ACOT12* (Supplementary Figure S4). Conversely, two previously deemed insignificant genes, *TLL1* and *CYP24A1*, achieved statistical significance. These findings indicate that incorporating missense and rare non-PathVar SNVs weakened the relationship between PathVar SNVs and HM.

**Figure 5:**
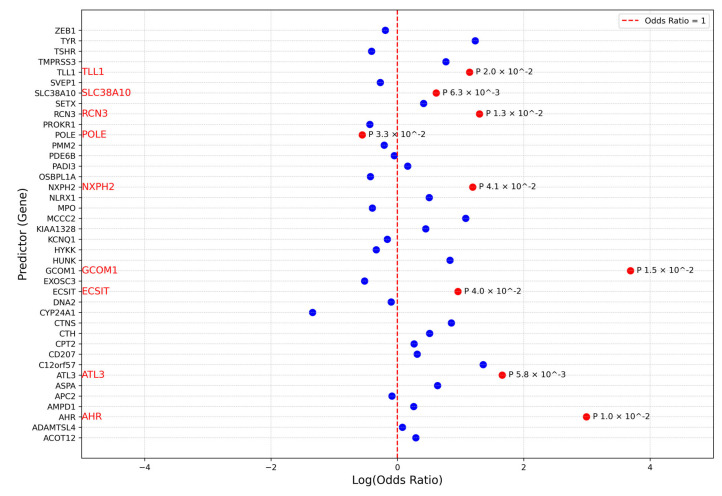
This figure presents the results of the standard logistic regression analysis, which included PathVar SNVs and rare missense variants with AF < 0.01 as qualifying variants. The analysis provides insights into the direction of the relationship between the priotirised genes and the case-cohort. The *Y*-axis lists the genes as predictors, while the *X*-axis represents the beta coefficients. The red dotted line, corresponding to an Odds Ratio of 1, separates the positive and negative sides of the logarithmic scale of the Odds Ratios. Red dots indicate beta coefficients with significant *p*-values, while blue dots represent non-significant beta coefficients. Genes with statistically significant associations are highlighted in red, whereas those without significant associations are depicted in blue.

In our analysis, we utilised the SKAT package in R to conduct burden testing, modelling the effect of each gene in the genotype matrix against the phenotype column in the dataset. The results from the second strategy revealed that many genes exhibited significant associations with the phenotype, suggesting a statistically significant relationship between gene effects in the genotype matrix and HM. To further clarify these associations, we applied a standard logistic regression model (Figure 5). This approach allowed us to explore the relationship between significant genes and the case class in the phenotype column by estimating odds ratios to quantify effect sizes and beta coefficients to assess the direction of these relationships. It is important to note that these interpretations are statistical in nature and do not necessarily reflect biological mechanisms. The gene *TYR* was deemed insignificant, as its *p*-values from the Burden test were close to 0.05 (0.040). Meanwhile, *POLE* remained significant, and *CYP24A1* became significant (Supplementary Figure S3), but both showed a negative beta coefficient (Figure 5), indicating an inverse relationship with HM.

**Figure 6:**
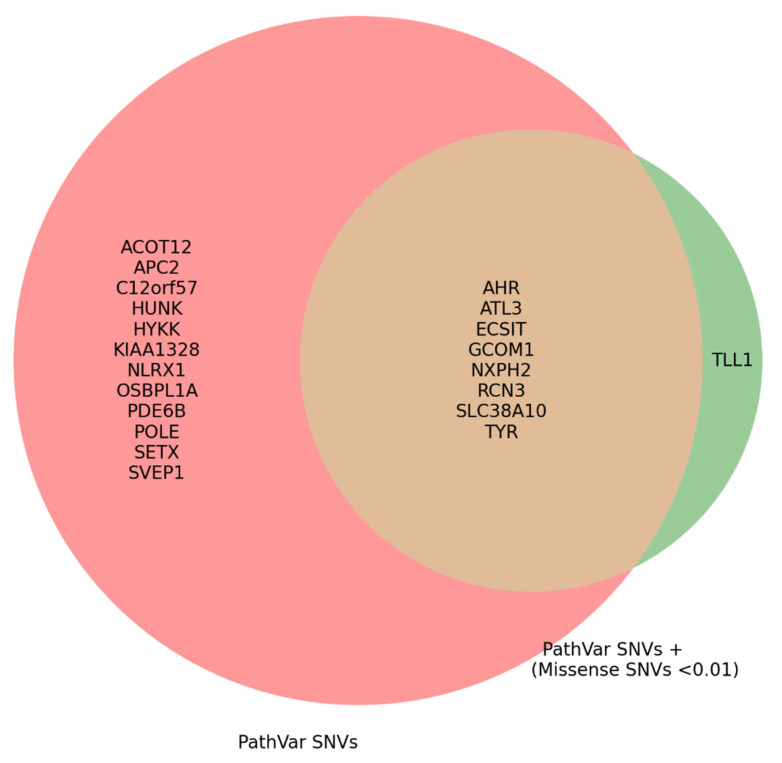
This figure illustrates the total significant genes potentially associated with HM, identified through two complementary strategies. Each circle represents a distinct approach for incorporating qualifying SNVs. The larger red circle denotes the first strategy, utilising SKAT_Burden alone, while the smaller circle represents the second strategy, combining SKAT_Burden with logistic regression.

The inclusion of non-PathVar SNVs in the second strategy further diluted the relationship between PathVar SNVs and HM, weakening the associations observed in the first strategy. It appears that, as we move from including only PathVar SNVs to combining PathVar with missense and rare SNVs, the strength of the relationship progressively diminishes. The overlapping genes identified in both strategies, which potentially contribute to HM, are shown in Figure 6. This is also clear from the proportions of qualifying SNVs for the shared genes, as detailed in Table 2.

## 4. Discussion

Our study revealed that population stratification exists within our dataset but does not significantly confound the relationship between genetic variants and HM. Using PCA and UMAP, we captured and adjusted for this stratification effectively. Burden testing identified several genes, including *GCOM1*, *SETX*, and *SLC38A10*, as significantly associated with HM, many of which have known roles in neurological function or migraine-related pathways. An expanded variant inclusion strategy confirmed the robustness of many associations and highlighted the complementary value of SKAT and logistic regression in genetic association analysis.

In case–control study designs, a paramount challenge lies in the investigation of confounding variables, which have the potential to mask the true relationship between predictor variables and the outcome of interest. A common practice in this context involves adjusting for both known and unknown factors that intersect the pathway between the predictors under examination and the outcome variable. Notably, in genetic studies, comprehensive clinical and demographic data are often limited, prompting researchers to adjust for unknown predictors through either study design or mathematical methodologies.

Controlling for unknown factors by design in genetic studies necessitates prospective participant recruitment, a frequently costly and logistically challenging process. In response to these constraints, PCA has emerged as a prevalent approach in both population and clinical genetic studies, employed to detect population stratification that may differentiate between cases and controls [75]. Although PCA does have its limitations [76], it remains a valuable approach for capturing evidence of population stratification independent of disease status.

Our initial investigation utilising PCA revealed evidence of population stratification, a finding anticipated given the diversity of genetic backgrounds among populations and individuals within those populations. This evidence is further supported by the UMAP technique, which reduced our 20 PCs and projected them into two dimensions.

The primary objective thereafter became capturing any mathematical dimentionality in the datasets that may represent true differences between cases and controls in the general population independent of disease status. Following a quality control process, our final subset of SNVs captured population stratification independent of disease. In all association analyses conducted, adjustments were made for the top four PCs, as they explain the majority of variance in our dataset and are likely to capture differences between cases and controls including, potentially, ancestral differences, consistent with the nature of top PCs [72].

The adjustment for PCs and other covariates yielded minimal differences in *p*-value numbers and a few genes significance level changes, which can be interpreted to suggest that the population stratification in our dataset, although it exists, does not confound the relationship between the genes tested and HM. This outcome underscores the efficacy of our methodological approach in mitigating the impact of population stratification on the observed associations, thereby enhancing the validity of our findings.

Our previous burden testing analysis revealed an increase in the burden of missense variants in the *CACNA1H* and *CACNA1l* genes using the current cohort [16]. Our current burden testing, which expands the investigation to include all potentially implicated HM genes, revealed that many genes were significantly associated with HM when the qualifying variants included only PathVar SNVs using SKAT. Notably, several of these genes have been implicated in neurological conditions and may potentially contribute to the development of HM, including *GCOM1* [69], *SETX* [70], and *SLC38A10* [71]. These genes were associated with the known FHM genes and were ranked based on their relevance to the FHM genes (Supplementary Table S2). The identification of these genes is consistent with the theory that multiple neurological conditions may share a common underlying genetic architecture [77,78].

The *SETX* gene, for example, has been implicated in the pathogenesis of two distinct neurological conditions, namely ataxia with oculomotor apraxia type 2 (AOA2) [79,80] and amyotrophic lateral sclerosis type 4 (ALS4) [70]. Strong evidence suggests that pathogenic variants within this gene exert a profound influence on the intricate relationship between immune response mechanisms and the development of these conditions. Although a direct causal link between the *SETX* gene and HM has not been established, it is conceivable that this gene may play a contributory role in the pathogenesis of HM by modulating immune response pathways, thereby warranting further investigation into its potential implications.

The *SLC38A10* gene has emerged as a potential candidate HM gene due to its role in disrupting the homeostasis of neurotransmitters, particularly glutamate [71]. As a member of the *SLC38* family, this gene is involved in regulating protein synthesis and cellular stress responses. Notably, glutamate is a pivotal neurotransmitter regulator, with a well-established link to migraine pathophysiology. A substantial body of evidence [81,82] suggests that glutamate excitotoxicity can trigger migraines, highlighting the importance of glutamate regulation in maintaining neurological homeostasis. The *SLC38A10* gene, which transports glutamate in a manner that confers protection against stress and glutamate toxicity [83], is, therefore, a critical component in modulating migraine susceptibility. Variants that disrupt the function of this transporter may consequently influence an individual’s predisposition to migraines, underscoring the need for further research into the role of *SLC38A10* in HM pathogenesis.

While a direct connection between *GCOM1* and migraine has not yet been confirmed, its association with the ion channel NMDA receptors and the involvement of glutamate in migraine pathology present compelling possibilities. Specifically, *GCOM1* interacts with the NR1 subunit of NMDA receptors, which play a pivotal role in migraine development, particularly in CSD, a key mechanism behind migraine aura [84]. Studies have demonstrated that NMDA receptor antagonists can effectively inhibit the initiation and spread of CSD, underscoring the significance of NMDA receptor activation in this process [85]. Moreover, NMDA receptors are involved in both peripheral and central neuronal sensitisation, a process closely tied to allodynia, a frequent migraine symptom [84]. Although *GCOM1* has not been directly studied in this context, its relationship with NMDA receptors implies it could indirectly influence migraine susceptibility. Adding to this possibility, glutamate is a major contributor to neurogenic inflammation and the activation of the trigeminal pain pathway, both essential elements of migraine pathology. Considering *GCOM1*’s interaction with NMDA receptors, it may represent an understudied factor in the intricate genetic framework of the migraine [86].

In the second strategy of our association analysis, we expanded the list of qualifying variants to include missense and rare SNVs, adopting a complementary approach to investigate the relationship between the prioritised genes and HM. The expanded analysis yielded a smaller list of genes with significant *p*-values, with the total number decreasing from 20 to 11. The addition of missense and rare SNVs clearly diluted the strength of the association between PathVar SNVs and HM. Notably, 9 genes that were deemed significant in the first strategy remained significant, while 2 new genes emerged as significant.

We used standard logistic regression to examine the direction of the significant relationships between genes from the second strategy and HM. Twelve genes were significant, but *POLE* and *CYP24A1*, which were significant in SKAT, had negative beta coefficients, indicating an inverse association with HM. This inverse relationship was not evident from SKAT analysis, highlighting the need for Odds Ratios and beta coefficients for clarification. Only two genes, *TYR* and *CYP24A1*, were rendered insignificant by the logistic regression. This is understandable as the difference in *p*-values between logistic regression (Wald test) and SKAT stems from their distinct approaches [54]. Logistic regression evaluates the individual effect of a variant, adjusting for covariates, and is sensitive to sample size and variant frequency. SKAT uses a kernel-based method to assess a variant in the context of others, making it more robust to rare variants and small samples. Logistic regression tests individual effects, while SKAT aggregates effects, leading to variations in *p*-values due to differing methods and assumptions.

### Limitations and Strengths

Our research has a few limitations. First, the PathVar pipeline utilises the Tapes tool, which attempts to apply the ACMG criteria as accurately as possible. However, applying these criteria is not 100% accurate, which may introduce some degree of uncertainty into our results. As is often the case in genetic studies, clinical information about both cohorts is limited. This lack of information may restrict our ability to fully interpret the results and identify potential correlations between genetic variants and HM. Additionally, while PCA is a common practice in genetic studies, it is not without limitations. PCA attempts to correct for ancestral differences and other unknown differences represented by the genotype information in our datasets. However, this method may not fully account for all sources of variation, potentially introducing some degree of bias into our results.

The association analysis demonstrated that PathVar SNVs, regardless of their pathogenicity assignment, are unique to the case cohort. This finding suggests that PathVar enabled us to capture SNVs that explain the genetic diversity of HM, at least in the genes prioritised for association analysis. The use of standard logistic regression and burden testing with SKAT provided a comprehensive understanding of the genetic associations. Although these methods employ different *p*-value calculations and statistical assumptions, they yielded consistent results, thereby strengthening the validity of our findings. Our results align with the evolutionary perspective that rare diseases, such as HM, are often caused by rare and probably damaging variants. This consistency provides additional support for the understanding that PathVar SNVs are important contributors to the genetic architecture of HM. These strengths highlight the value of using PathVar to identify unique SNVs. PathVar’s ability to capture SNVs distinctive to the case cohort underscores its utility in identifying genetic variants potentially contributing to HM. Combining multiple analytical approaches, such as logistic regression and burden testing, enhances the understanding of genetic associations and bolsters confidence in the findings. Furthermore, integrating evolutionary perspectives on rare diseases adds valuable context to elucidating the genetic basis of HM and other complex disorders.

## 5. Conclusions

In this research, the second strategy sequentially reduced the number of significant genes from 20 to 11, offering a more refined understanding of the genetic landscape underlying HM. This reduction highlights the unique contribution of PathVar SNVs, which appeared more distinctive in the case cohort and were capable of capturing HM’s genetic architecture more effectively than in the controls. Notably, several genes remained significant even after incorporating missense and rare SNVs, reinforcing their potential involvement in HM.

Among the identified genes, novel candidates such as SLC38A10, GCOM1, and SETX, which were previously unassociated with HM, emerged as strong contributors, representing a significant advancement in the field. These findings suggest that PathVar SNVs may be more closely linked to functional changes relevant to HM pathology.

The iterative refinement of associations across the two variant strategies underscores the value of a multi-faceted approach in gene prioritisation. By considering distinct variant types, we gain a deeper insight into the complex genetic underpinnings of HM. This study not only broadens the spectrum of genes potentially involved in HM but also lays the groundwork for future functional studies and clinical applications, ultimately advancing our understanding of this rare neurological disorder.

## Figures and Tables

**Table 1:** Different rare variant association tests.

Method	Type	Key Features	Advantages	Limitations
Burden Tests	Collapsing	Aggregates rare variants into a single score for analysis	High power when all variants affect trait in same direction	Loses power with bidirectional or non-causal variants
SKAT (Sequence Kernel Association Test)	Kernel-based	Models distribution of variant effects; allows covariate adjustment	Handles bidirectional effects; flexible modeling	Lower power if all variants affect in same direction
SKAT-O	Omnibus (Hybrid)	Combines SKAT and burden tests using Fisher’s method	Balances power across different genetic architectures	May lose power when few trait-associated variants exist
C-alpha Test	Distribution-based	Tests for variability in effect direction among variants	Detects both risk-increasing and protective variants	Lower power when effects are unidirectional
SSU Test (Sum of Squared Score)	Distribution-based	Captures total variance in genetic effects	Useful for mixed-direction effects	Sensitive to number of causal variants
KBAC (Kernel-Based Adaptive Cluster)	Kernel-based	Clusters similar genotypes; adaptive weighting	Effective for complex genotype-phenotype relationships	Computationally intensive
CMC (Combined Multivariate and Collapsing)	Hybrid	Combines rare and common variants; uses Hotelling’s T^2^ test	Incorporates broad variant spectrum	Assumes consistent direction of effect
ACAT (Aggregated Cauchy Association Test)	*p*-value Combination	Combines *p*-values using Cauchy distribution	Good power when few strong-effect variants are present	May underperform with many weak signals
Sub-regional Collapsing	Collapsing	Targets functionally intolerant genomic sub-regions	Enhances detection of clustered pathogenic variants	Requires accurate regional intolerance annotation

**Table 2:** Significant genes shared by both strategies.

PathVar SNVs + (Missense SNVs < 0.01)							
CHR	Gene	SKAT_Burden			Logistic Regression		
		Cases SNVs%	Controls SNVs%	Buden_Pvalue	Odds Ratio	log_Pvalue	Coefficient
2	NXPH2	0.02	0.007	0.02	3.29	0.04	1.18
7	AHR	0.01	0.001	0.004	19.93	0.009	2.99
11	ATL3	0.02	0.005	0.004	5.25	0.005	1.65
11	TYR	0.02	0.006	0.04	3.42	0.05	1.23
15	GCOM1	0.01	0	0.002	39.94	0.01	3.68
17	SLC38A10	0.10	0.076	0.006	1.85	0.006	0.61
19	ECSIT	0.03	0.01	0.02	2.60	0.03	0.95
19	RCN3	0.03	0.01	0.01	3.66	0.01	1.29

## Data Availability

The datasets generated during and/or analysed during the current study are available from the corresponding author on reasonable request.

## Supplementary Materials

The following supporting information can be downloaded at: https://www.mdpi.com/article/10.3390/genes16070807/s1, Figure S1: This figure illustrates the functional classification of prioritised genes implicated in neurological processes. Genes are grouped into five categories based on their known or predicted biological roles according to gene ontology annotations, with each category distinguished by a unique colour. The Ion Channels & Transporters category (red) includes genes involved in ion transport and membrane channel activity, such as *KCNQ1* and *SLC38A10*. The Neural Synapses & Neurotransmission group (teal) consists of genes related to synaptic structure, neurotransmission, and neuronal connectivity, including *NXPH2* and *APC2*. The Neurohormones & Neuroendocrine category (yellow) contains genes like *AHR* and *TSHR*, which are involved in hormonal signalling and neuroendocrine regulation. Genes associated with cerebrovascular integrity and the blood–brain barrier (BBB), such as *SVEP1* and *MPO*, are classified under Brain Vasculature & BBB (green). Finally, genes not fitting these categories but involved in various metabolic, structural, or regulatory roles, such as *ZEB1*, *POLE*, and *PMM2*, are grouped under Other (Metabolic/Structural) (purple). The tree-like structure and colour coding provide a clear visual summary of the functional diversity of the genes analysed; Figure S2: An initial investigation of population stratification was conducted, as illustrated in this figure, which plots the first PC on the *X*-axis and the second PC on the *Y*-axis. The data points are colour-coded based on phenotype: green dots represent individuals diagnosed with HM. In contrast, red dots correspond to control subjects with no reported history of neurological conditions; Figure S3: This UMAP plot provides clear evidence of population stratification within the dataset. The figure represents the dimensionality reduction of 20 PCs into two dimensions, with the first dimension plotted on the *X*-axis and the second on the *Y*-axis. Data points are colour-coded according to phenotype: green dots indicate individuals with HM, while red dots represent the control cohort; Figure S4: This figure presents the results of the second burden testing analysis, in which missense and rare SNVs with an AF < 0.01 were added to the list of qualifying variants. PathVar genes were modelled against the phenotype using the SKAT package in R. The *Y*-axis represents the *p*-value on a logarithmic scale, and the *X*-axis corresponds to the genes. The dotted line denotes the significance threshold, with red dots indicating significant *p*-values and blue dots representing non-significant *p*-values. Significant genes are highlighted in red, while non-significant genes are shown in blue; Table S1: This table lists the full list of qualifying PathVar SNVs analysed in the first strategy; Table S2: This table ranks the tested genes based on their relevance to the known FHM genes.

## Abbreviations

The following abbreviations are used in this manuscript frequently:

HM	Hemiplegic Migraine
WES	Whole Genome Sequencing
PCA	Principal Component Analysis
SNVs	Single Nucleotide Variants
MA	Migraine with Aura
MO	Migraine without Aura
FHM	Familial Hemiplegic Migraine
MAF	Minor Allele Frequency
GWASs	Genome-Wide Association Studies
NGS	Next-Generation Sequencing
ACMGs	American College of Medical Genetics
PCs	Principal Components
