# Supplementary material for: Gene-Based Burden Testing of Rare Variants in Hemiplegic Migraine: A Computational Approach to Uncover the Genetic Architecture of a Rare Brain Disorder
Source: Genes (Basel). 2025 Jul 9;16(7):807. doi: 10.3390/genes16070807 (PMC12294318; doi:10.3390/genes16070807)
Supplement: Supplementary file 1 [file genes-16-00807-s001.zip › Alfayyadh.etal.Supplementary-materials.pdf]

SUPPLEMENTARY MATERIALS

Ion Channels & Transporters

```
├── KCNQ1
├── CTNS
├── SLC38A10
├── PDE6B
└── CPT2
```

Neural Synapses & Neurotransmission

```
├── NXPH2
├── PROKR1
├── APC2
├── PADI3
└── SETX
```

Neurohormones & Neuroendocrine

```
├── AHR
├── TSHR
├── TYR
└── CYP24A1
```

Brain Vasculature & Blood Brain Barrier

```
├── SVEP1
├── ADAMTSL4
└── MPO
```

Other (Metabolic/Structural)

```
├── AMPD1
├── CTH
├── RCN3
├── NLRX1
├── HUNK
├── ECSIT
├── KIAA1328
├── OSBPL1A
├── HYKK
├── CD207
├── GCOM1
├── C12orf57
├── POLE
├── ZEB1
├── DNA2
├── EXOSC3
├── MCCC2
├── TLL1
├── TMPRSS3
├── ACOT12
├── ASPA
├── PMM2
└── ATL3
```

Figure S1. This figure illustrates the functional classification of prioritised genes implicated in neurological processes. Genes are grouped into five categories based on their known or predicted biological roles according to gene ontology annotations, with each category distinguished by a unique colour. The Ion Channels & Transporters category (red) includes genes involved in ion transport and membrane channel activity, such as ***KCNQ1*** and ***SLC38A10***. The Neural Synapses & Neurotransmission group (teal) consists of genes related to synaptic structure, neurotransmission, and neuronal connectivity, including ***NXPH2*** and ***APC2***. The Neurohormones & Neuroendocrine category (yellow) contains genes like ***AHR*** and ***TSHR***, which are involved in hormonal signalling and neuroendocrine regulation. Genes associated with cerebrovascular integrity and the blood–brain barrier (BBB), such as ***SVEP1*** and ***MPO***, are classified under Brain Vasculature & BBB (green). Finally, genes not fitting these categories but involved in various metabolic, structural, or regulatory roles, such as ***ZEB1, POLE***, and ***PMM2***, are grouped under Other (Metabolic/Structural) (purple). The tree-like structure and colour coding provide a clear visual summary of the functional diversity of the genes analysed.

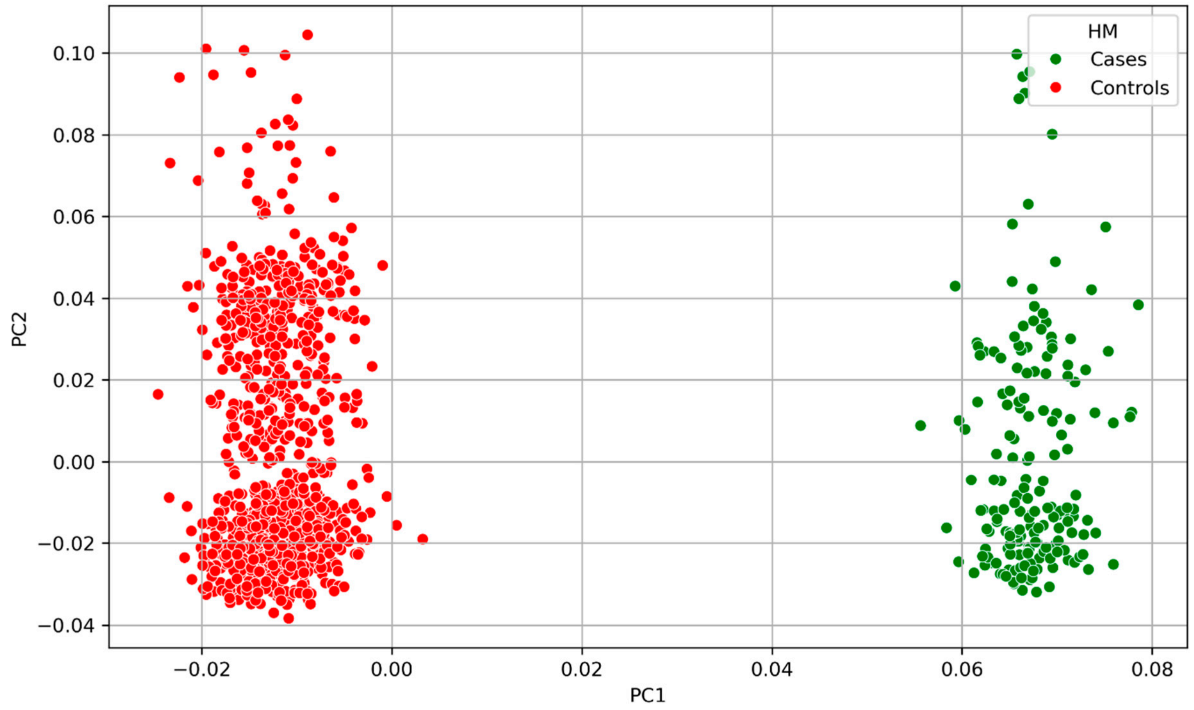

Figure S2. An initial investigation of population stratification was conducted, as illustrated in this figure, which plots the first PC on the X-axis and the second PC on the Y-axis. The data points are colour-coded based on phenotype: green dots represent individuals diagnosed with HM. In contrast, red dots correspond to control subjects with no reported history of neurological conditions.

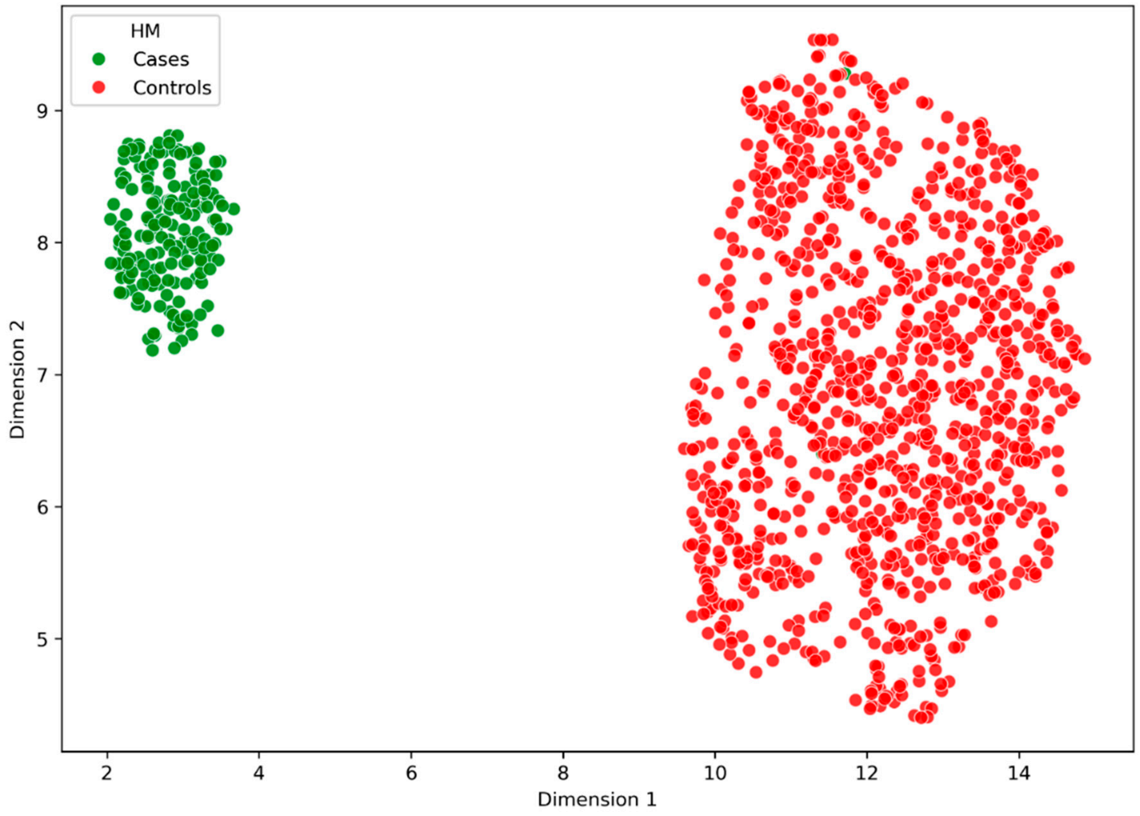

Figure S3. This UMAP plot provides clear evidence of population stratification within the dataset. The figure represents the dimensionality reduction of 20 PCs into two dimensions, with the first dimension plotted on the X-

axis and the second on the Y-axis. Data points are colour-coded according to phenotype: green dots indicate individuals with HM, while red dots represent the control cohort.

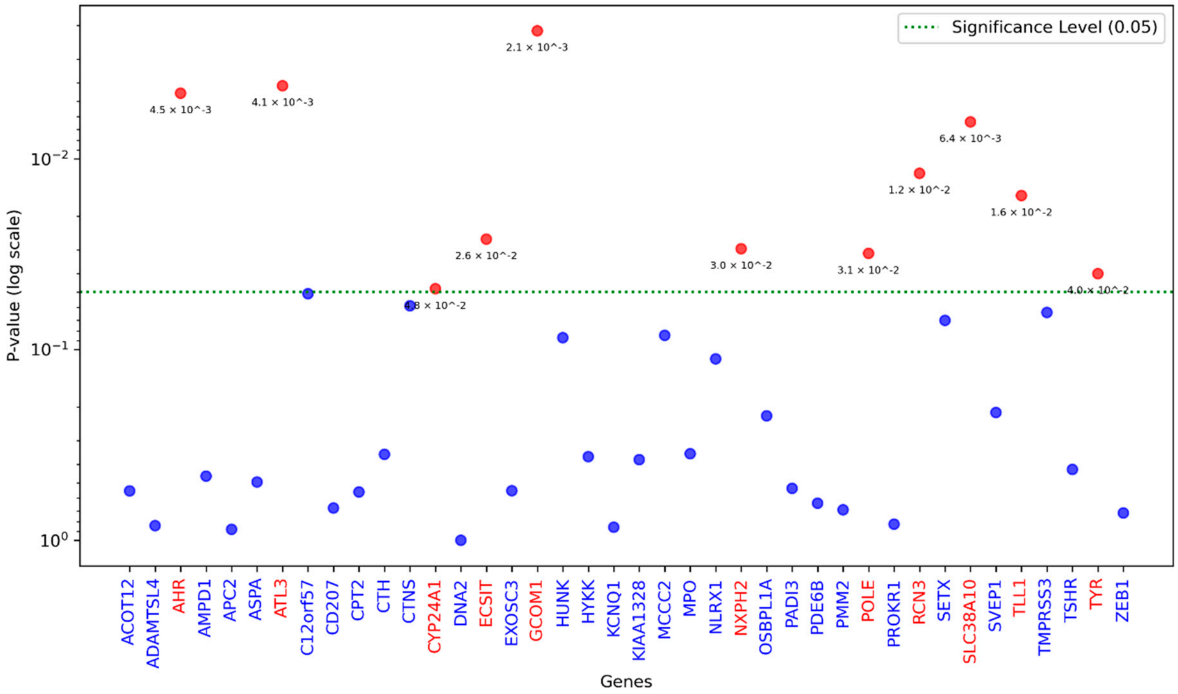

Figure S4. This figure presents the results of the second burden testing analysis, in which missense and rare SNVs with an AF < 0.01 were added to the list of qualifying variants. PathVar genes were modelled against the phenotype using the SKAT package in R. The Y-axis represents the P-value on a logarithmic scale, and the X-axis corresponds to the genes. The dotted line denotes the significance threshold, with red dots indicating significant P-values and blue dots representing non-significant P-values. Significant genes are highlighted in red, while non-significant genes are shown in blue.
